# Editorial: HIV and Cancer Immunotherapy: Similar Challenges and Converging Approaches

**DOI:** 10.3389/fimmu.2020.00519

**Published:** 2020-03-31

**Authors:** Mirko Paiardini, Kavita Dhodapkar, Justin Harper, Steven G. Deeks, Rafi Ahmed

**Affiliations:** ^1^Division of Microbiology and Immunology, Yerkes National Primate Research Center, Emory University, Atlanta, GA, United States; ^2^Department of Pathology and Laboratory Medicine, Emory University School of Medicine, Atlanta, GA, United States; ^3^Department of Pediatrics, Emory University School of Medicine and Children's Healthcare of Atlanta, Atlanta, GA, United States; ^4^Division of Experimental Medicine, University of California, San Francisco, San Francisco, CA, United States; ^5^Emory Vaccine Center and Department of Microbiology and Immunology, Emory University School of Medicine, Atlanta, GA, United States

**Keywords:** HIV, cancer, ART, immunotherapy, immune surveillance, immune checkpoint blockade, inflammation, CAR T cells

## Introduction

Although modern anti-retroviral therapy (ART) permits near-normal life expectancies by suppressing viral replication to clinically undetectable levels in people living with HIV (PLWH) (1), sustained treatment is complicated by complex pharmacological (i.e., adverse events, adherence, resistance) and societal issues (i.e., stigma, cost burden, medical access). Furthermore, ART is incapable of eliminating the latent viral reservoir, which is responsible for recrudescence when therapy is interrupted (2–5). Viral persistence is facilitated by a variety of mechanisms such as the exhaustion of HIV-specific cytolytic T-cells (CTLs) driven by chronic inflammation (6–8); epigenetic modifications to dampen the expression of viral proteins allowing evasion of immunosurveillance (9, 10); the localization of infected cells within immune privileged anatomical sites (11–13); and the survival of long-lived, virus-harboring cells allowing reservoir expansion via homeostatic proliferation (14, 15). Although formidable challenges exist for completing eradicating HIV from infected individuals (a “cure”), there is growing enthusiasm that novel immunotherapy approaches might eventually result in durable control of replication-competent HIV in absence of any therapy (a “remission”). Much of this enthusiasm comes from dramatic progress made in using immunotherapy to treating cancer. This editorial summarizes how the 13 review articles included in this special issue highlight key parallels between HIV and tumor persistence as well as how these similarities inform the development of novel immunotherapy-based strategies toward an HIV cure.

## The Persistence of Memory

In both HIV and cancer, subsequent pathology arises from a relatively rare, yet difficult to distinguish and persistent subset of cells. In the non-human primate model of HIV infection, the persistent viral reservoir is established within 4–9 days post-infection (16); similarly, very early ART initiation does not induce viral remission in PLWH (17). In a meta-analysis of human cohorts, Etemad et al. propose that preferential infection of transitional memory (T_TM_) CD4^+^ T-cells, as opposed to longer-lived central memory or naïve cells, is a key predictor for post-treatment control (18) despite weak HIV-specific CD8^+^ T-cell responses. Intriguingly, Goonetilleke et al. hypothesize that the generation of the long-lived reservoir, particularly in central memory (T_CM_) and stem-cell memory (T_SCM_) CD4^+^ T-cells, can be blunted by inhibiting the IL-7 signaling axis, thereby disrupting the transition and maintenance of CD127^+^ memory subsets from highly-infected effector CD4^+^ T-cells (18). Gavegnano et al. explore the use of Jak inhibitors in inhibiting the activity of the anti-apoptotic Bcl-2 protein to reduce cellular lifespans (19, 20). By blocking the formation and maintenance of the viral reservoir in long-lived memory subsets, the authors proposed that a reduction in viral burden will facilitate HIV remission as mimicked in post-treatment controllers.

## Escape Through Editing

Once the viral reservoir is established, HIV-specific CD8^+^ T-cells are required for viral suppression (21, 22); however, in most infected people, HIV-specific CTLs are incapable of eliminating infected cells (23) indicative of failure in immune surveillance independent of mutational escape or dysfunction (24). This incomplete elimination permits subsequent equilibrium phase sculpting of reservoir-harboring cells by immune pressures, which in cancer models has been termed “immunoediting” (25). Analogous to “antigen loss” in tumors models, Huang et al. explore the novel concept that during ART cells harboring replication-competent virus undergo clonal expansion with subsequent immunoediting; thereby decreasing CTL susceptibility by selecting for BCL-2 expression (26) and integration sites favoring cell division (27, 28). As HIV infection impacts on cellular metabolism and oxidative stress (29, 30), immunoediting may also select for an altered cellular lipid antigen composition that, as summarized by Tiwary et al., in oncology models impinges on chronic inflammation by modulating the macrophage M1 to M2 balance (31) and impairs antigen processing in dendritic cells (32); specifically, CD1d antigen loading for natural killer T-cells (NKT) (33). As a model comparison (Mota and Jones) examine how HTLV-1 generates malignant “repliclones” by an interplay of host- and viral-mediated immunoediting. Therefore, these articles support the notion that HIV CTL escape might be more complex than viral epitope mutations, but rather involve the progressive selection of immunoedited, infected cells resistant to immune surveillance.

## Who Watches the Watchmen?

Effective immunosurveillance of HIV-infected cells remains problematic as CTLs exhibit exhausted effector functions arising from chronic inflammation and antigen persistence during the natural course of infection and residual inflammation, driven by microbial translocation in the gut, despite suppressive ART (34, 35). Structural defects in gut integrity cause by HIV further impacts the microbiota distribution (36), which given its ability in cancer models to modulate toxicity (37) and therapy efficacy (38, 39), may represent an attractive therapeutic avenue as proposed by Herrera et al.. In some respects, as describe by Dhodapkar and Dhodapkar, ART-suppressed HIV mirrors pre-clinical malignancy, a prolonged state characterized by early-onset of T-cell exhaustion coupled with the depletion of stem cell memory (40). However, unlike antigen-rich tumor models, curative HIV therapies require that latent virus be reactivated to render infected cells immunogenic and cleared by potent anti-HIV CTLs (“kick and kill”) (10, 41). Given their capacity to promote tumor clearance, as detailed by Puronen et al., many immunotherapies are being investigated in HIV cure studies to induce T-cell activation and restore CTL functionality, such anti-PD-1 and anti-CTLA-4 check point inhibitors (CPI) (42–44), and IL-7 and IL-15 cytokine therapy (45, 46). Given emerging data concerning the importance of innate natural killer (NK) cells in the control of HIV and cancers (47, 48), Lucar et al., discuss immunotherapies targeting NKG2a and killer-cell immunoglobulin-like receptors (KIRs) as novel strategies to determine whether dysfunction NK cell states can be rescued. Curative strategies centered around CPIs have revolutionized the treatment of certain refractory cancers by reinvigorating the host immune response; yet, in PWLH it remains to be seen whether antigen burden is a critical determinant of response.

## In Case of Emergency—Break Glass

Beyond these strategies, which may above prove too toxic, fail to penetrate tissue, or lack desire specificity, alternative curative approaches utilize adoptive T-cell therapy to redirect CTL responses. Kim et al. describe the re-emergence of chimeric antigen receptor (CAR) T-cells as an attractive immunotherapy strategy given its progressive re-engineering in oncology settings to improve safety, expression, and persistence (49). Although CAR T-cells have attained remarkable remission rates for CD19^+^ B-cell acute lymphoblastic leukemia (50), significant relapse rates are associated with diminished persistence upon antigen loss/escape, the suppressive tumor microenvironment, and impaired tumor penetration (51). These issue impacting tumor relapse are directly analogous to HIV models vis-á-vis ART-mediated aviremia, the expansion of regulatory T-cells (T_REGs_) (52, 53), and the exclusion of CTLs from secondary lymphoid tissue (13, 54). Possible strategies to surmount these issues include engineering CAR T-cells to express 4-1BB co-stimulatory domains allowing oxidative metabolism (55); secrete cytokines, such as IL-12 or IL-18 (56, 57); and up-regulate the chemokine receptor CXCR5 to promote homing to the lymphoid B-cell follicle (58) as explored by Mylvaganam et al.. Seemingly, CAR T-cells for HIV applications should be directed against viral proteins to minimize safety concerns and given the lack of reliable biomarkers to identify latently-infected cells. Ergo, CAR T-cells will likely require co-administration with potent latency reactivating agents to promote therapy persistence and reveal cellular targets for clearance. Such combination therapies would benefit from positron emission tomography (PET)-based imaging, as reviewed by Henrich et al., to observe the total-body viral antigen distribution (59, 60) and to gain insights concerning the potential for efficacy in difficult to sample tissues (61, 62).

## Summary

Models of cancer and HIV persistence share an interesting paradox: responses promoting self-tolerance when exposed to sustained inflammatory stimuli permit pathological dissemination and escape from immune surveillance. This similarity would suggest common curative approaches via the targeting of immunosuppressive pathways. However, a key distinction is that in cancer the self-immunogen is pervasive; whereas, in ART-treated HIV infection chronic antigenic stimulation arises largely from gut microbial translocation, not from viral proteins. This different in antigen source may represent a key obstacle when translating therapies between cancer and HIV models (63). In designing immunotherapy strategies, it is also important to consider that adverse event outcomes between these models have substantially different tolerances, as HIV is a manageable chronic disease and cancers are invariably fatal. Future trials will be necessary to determine whether these mechanistic insights regarding escape and exhaustion can be successfully adapted to facilitate long-term, ART-free HIV remission.

## Author Contributions

MP, KD, SD, and RA contributed to formulating the theme for this article collection, recruiting authors, and acting as editors for the submissions. MP and JH wrote the editorial, with contributions, and final edits from all authors.

### Conflict of Interest

The authors declare that the research was conducted in the absence of any commercial or financial relationships that could be construed as a potential conflict of interest.
